# Sparse partial least squares regression for simultaneous dimension reduction and variable selection

**DOI:** 10.1111/j.1467-9868.2009.00723.x

**Published:** 2010-01

**Authors:** Hyonho Chun, Sündüz Keleş

**Affiliations:** University of WisconsinMadison, USA

**Keywords:** Chromatin immuno-precipitation, Dimension reduction, Gene expression, Lasso, Microarrays, Partial least squares, Sparsity, Variable and feature selection

## Abstract

Partial least squares regression has been an alternative to ordinary least squares for handling multicollinearity in several areas of scientific research since the 1960s. It has recently gained much attention in the analysis of high dimensional genomic data. We show that known asymptotic consistency of the partial least squares estimator for a univariate response does not hold with the very large *p* and small *n* paradigm. We derive a similar result for a multivariate response regression with partial least squares. We then propose a sparse partial least squares formulation which aims simultaneously to achieve good predictive performance and variable selection by producing sparse linear combinations of the original predictors. We provide an efficient implementation of sparse partial least squares regression and compare it with well-known variable selection and dimension reduction approaches via simulation experiments. We illustrate the practical utility of sparse partial least squares regression in a joint analysis of gene expression and genomewide binding data.

## 1. Introduction

With the recent advancements in biotechnology such as the use of genomewide microarrays and high throughput sequencing, regression-based modelling of high dimensional data in biology has never been more important. Two important statistical problems commonly arise within regression problems that concern modern biological data. The first is the selection of a set of *important* variables among a large number of predictors. Utilizing the sparsity principle, e.g. operating under the assumption that a small subset of the variables is deriving the underlying process, with *L*_1_-penalty has been promoted as an effective solution (Tibshirani, 1996; Efron *et al.*, 2004). The second problem is that such a variable selection exercise often arises as an ill-posed problem where

- the sample size *n* is much smaller than the total number of variables (*p*) and
- covariates are highly correlated.

Dimension reduction techniques such as principal components analysis (PCA) or partial least squares (PLS) have recently gained much attention for addressing these within the context of genomic data (Boulesteix and Strimmer, 2006).

Although dimension reduction via PCA or PLS is a principled way of dealing with ill-posed problems, it does not automatically lead to selection of relevant variables. Typically, all or a large portion of the variables contribute to final direction vectors which represent linear combinations of original predictors. Imposing sparsity in the midst of the dimension reduction step might lead to simultaneous dimension reduction and variable selection. Recently, Huang *et al.* (2004) proposed a penalized PLS method that thresholds the final PLS estimator. Although this imposes sparsity on the solution itself, it does not necessarily lead to sparse linear combinations of the original predictors. Our goal is to impose sparsity in the dimension reduction step of PLS so that sparsity can play a direct principled role.

The rest of the paper is organized as follows. We review general principles of the PLS methodology in Section 2. We show that PLS regression for either a univariate or multivariate response provides consistent estimators only under restricted conditions, and the consistency property does not extend to the very large *p* and small *n* paradigm. We formulate sparse partial least squares (SPLS) regression by relating it to sparse principal components analysis (SPCA) (Jolliffe *et al.*, 2003; Zou *et al.*, 2006) in Section 3 and provide an efficient algorithm for solving the SPLS regression formulation in Section 4. Methods for tuning the sparsity parameter and the number of components are also discussed in this section. Simulation studies and an application to transcription factor activity analysis by integrating microarray gene expression and chromatin immuno-precipitation–microarray chip (CHIP–chip) data are provided in Sections 5 and 6.

## 2. Partial least squares regression

### 2.1. Description of partial least squares regression

PLS regression, which was introduced by Wold (1966), has been used as an alternative approach to ordinary least squares (OLS) regression in ill-conditioned linear regression models that arise in several disciplines such as chemistry, economics and medicine (de Jong, 1993). At the core of PLS regression is a dimension reduction technique that operates under the assumption of a basic latent decomposition of the response matrix (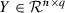) and predictor matrix (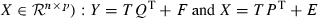, where 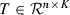 is a matrix that produces *K* linear combinations (scores); 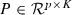 and 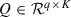 are matrices of coefficients (loadings), and 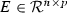 and 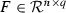 are matrices of random errors.

To specify the latent component matrix *T* such that *T*=*XW*, PLS requires finding the columns of *W*=(*w*_1_,*w*_2_,…,*w*_*K*_) from successive optimization problems. The criterion to find the *k*th direction vector *w*_*k*_ for univariate *Y* is formulated as 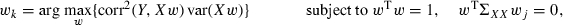
(1) for *j*=1,…,*k*−1, where Σ_*XX*_ is the covariance of *X*. As evident from this formulation, PLS seeks direction vectors that not only relate *X* to *Y* but also capture the most variable directions in the *X*-space (Frank and Friedman, 1993).

There are two main formulations for finding PLS direction vectors in the context of multivariate *Y*. These vectors were originally derived from an algorithm, known as NIPALS (Wold, 1966), without a specific optimization problem formulation. Subsequently, a statistically inspired modification of PLS, known as SIMPLS (de Jong, 1993), was proposed with an algorithm by directly extending the univariate PLS formulation. Later, ter Braak and de Jong (1998) identified the ‘PLS2’ formulation which the NIPALS algorithm actually solves. The PLS2 formulation is given by 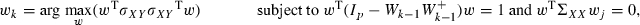
(2) for *j*=1,…,*k*−1, where *σ*_*XY*_ is the covariance of *X* and *Y*, *I*_*p*_ denotes a *p*×*p* identity matrix and 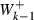 is the unique Moore–Penrose inverse of *W*_*k*−1_=(*w*_1_,…,*w*_*k*−1_). The SIMPLS formulation is given by 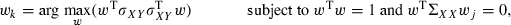
(3) for *j*=1,…,*k*−1. Both formulations have the same objective function but different constraints and thus yield different sets of direction vectors. Their prediction performances depend on the nature of the data (de Jong, 1993; ter Braak and de Jong, 1998). de Jong (1993) showed that both formulations become equivalent and yield the same set of direction vectors for univariate *Y*.

In the actual fitting of the PLS regression, either the NIPALS or the SIMPLS algorithm is used for obtaining the PLS estimator. The NIPALS algorithm produces the direction vector *d*_*k*+1_ with respect to the deflated matrix 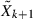 at the (*k*+1)th step by solving 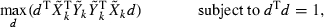 where 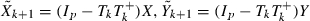 and 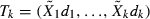. At the final *K*th step, 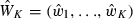, the direction matrix with respect to the original matrix *X*, is computed by 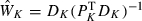, where 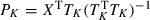 and *D*_*K*_=(*d*_1_,…,*d*_*K*_). In contrast, the SIMPLS algorithm produces the (*k*+1)th direction vector 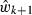 directly with respect to the original matrix *X* by solving 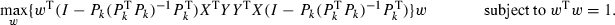


After estimating the latent components (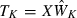) by using *K* numbers of direction vectors, loadings *Q* are estimated via solving min_*Q*_(‖*Y*−*T*_*KQ*_^T^‖_2_). This leads to the final estimator 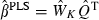, where 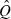 is the solution of this least squares problem.

### 2.2. An asymptotic property of partial least squares regression

#### 2.2.1. Partial least squares regression for univariate *Y*

Stoica and Soderstorom (1998) derived asymptotic formulae for the bias and variance of the PLS estimator for the univariate case. These formulae are valid if the ‘signal-to-noise ratio’ is high or if *n* is large and the predictors are uncorrelated with the residuals. Naik and Tsai (2000) proved consistency of the PLS estimator under normality assumptions on both *Y* and *X* in addition to consistency of *S*_*XY*_ and *S*_*XX*_ and the following condition 1. This condition, which is known as the Helland and Almoy (1994) condition, implies that an integer *K* exists such that exactly *K* of the eigenvectors of Σ_*XX*_ have non-zero components along *σ*_*XY*_.

*Condition 1*. There are eigenvectors *v*_*j*_ (*j*=1,…,*K*) of Σ_*XX*_ corresponding to different eigenvalues, such that 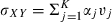 and *α*_1_,…,*α*_*K*_ are non-zero.

We note that the consistency proof of Naik and Tsai (2000) requires *p* to be fixed. In many fields of modern genomic research, data sets contain a large number of variables with a much smaller number of observations (e.g. gene expression data sets where the variables are of the order of thousands and the sample size is of the order of tens). Therefore, we investigate the consistency of the PLS regression estimator under the very large *p* and small *n* paradigm and extend the result of Naik and Tsai (2000) for the case where *p* is allowed to grow with *n* at an appropriate rate. In this setting, we need additional assumptions on both *X* and *Y* to ensure the consistency of *S*_*XX*_ and *S*_*XY*_, which is the conventional assumption for fixed *p*. Recently, Johnstone and Lu (2004) proved that the leading PC of *S*_*XX*_ is consistent if and only if *p*/*n*→0. Hence, we adopt their assumptions for *X* to ensure consistency of *S*_*XX*_ and *S*_*XY*_. Assumptions for *X* from Johnstone and Lu (2004) are as follows.

*Assumption 1*. Assume that each row of 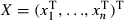 follows the model 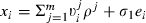, for some constant *σ*_1_, where

- *ρ*^*j*^,*j*=1,…,*m*≤*p*, are mutually orthogonal PCs with norms ‖*ρ*^1^‖≥‖*ρ*^2^‖≥…≥‖*ρ*^*m*^‖,
- the multipliers 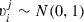 are independent over the indices of both *i* and *j*,
- the noise vectors *e*_*i*_∼*N*(0,*I*_*p*_) are independent among themselves and of the random effects 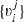 and
- *p*(*n*),*m*(*n*) and {*ρ*^*j*^(*n*),*j*=1,…,*m*} are functions of *n*, and the norms of the PCs converge as sequences: *ϱ*(*n*)=(‖*ρ*^1^(*n*)‖,…,‖*ρ*^*j*^(*n*)‖,…)→*ϱ*=(*ϱ*_1_,…,*ϱ*_*j*_,…). We also write *ϱ*_+_ for the limiting *l*_1_-norm: *ϱ*_+_=Σ_*j*_ *ϱ*_*j*_.

We remark that the above factor model for *X* is similar to that of Helland (1990) except for having an additional random error term *e*_*i*_. All properties of PLS in Helland (1990) will hold, as the eigenvectors of Σ_*XX*_ and 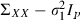 are the same. We take the assumptions for *Y* from Helland (1990) with an additional norm condition on *β*.

*Assumption 2*. Assume that *Y* and *X* have the relationship, *Y*=*Xβ*+*σ*_2_*f*, where 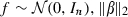 <∞, and *σ*_2_ is a constant.

We next show that, under the above assumptions and condition 1, the PLS estimator is consistent if and only if *p* grows much slower than *n*.

*Theorem 1*. Under assumptions 1 and 2, and condition 1,

- if *p*/*n*→0, then 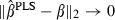 in probability and
- if *p*/*n*→*k*_0_ for *k*_0_>0, then 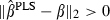 in probability.

The main implication of this theorem is that the PLS estimator is not suitable for very large *p* and small *n* problems in complete generality. Although PLS utilizes a dimension reduction technique by using a few latent factors, it cannot avoid the sample size issue since a reasonable size of *n* is required to estimate sample covariances consistently as shown in the proof of theorem 1 in Appendix A. A referee pointed out that a qualitatively equivalent result has been obtained by Nadler and Coifman (2005), where the root-mean-squared error of the PLS estimator has an additional error term that depends on *p*^2^/*n*^2^.

#### 2.2.2. Partial least squares regression for multivariate *Y*

There are limited or virtually no results on the theoretical properties of PLS regression within the context of a multivariate response. Counterintuitive simulation results, where multivariate PLS shows a minor improvement in prediction error, were reported in Frank and Friedman (1993). Later, Helland (2000) argued by intuition that, since multivariate PLS achieves parsimonious models by using the same reduced model space for all the responses, the net gain of sharing the model space could be negative if, in fact, all the responses require different reduced model spaces. Thus, we next introduce a specific setting for multivariate PLS regression in the light of Helland's (2000) intuition and extend the consistency result of univariate PLS to the multivariate case.

Assume that all the response variables have linear relationships with the *same* set of covariates: *Y*_1_=*Xb*_1_+*f*_1_,*Y*_2_=*Xb*_2_+*f*_2_,…,*Y*_*q*_=*Xb*_*q*_+*f*_*q*_, where *b*_1_,…,*b*_*q*_ are *p*×1 coefficient vectors and *f*_1_,…,*f*_*q*_ are independent error vectors from 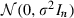. Since the shared reduced model space of each response is determined by *b*_*i*_s, we impose a restriction on these coefficients. Namely, we require the existence of eigenvectors *v*_1_,…,*v*_*K*_ of Σ_*XX*_ that span the solution space, which each *b*_*i*_ belongs to.

We have proved consistency of the PLS estimator for a univariate response using the facts that *S*_*XY*_ is proportional to the first direction vector and the solution space, which 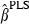 belongs to, can be explicitly characterized by 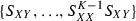. However, for a multivariate response, PLS finds the first direction vector as the first left singular vector of *S*_*XY*_. The presence of remaining directions in the column space of *S*_*XY*_ makes it difficult to characterize the solution space explicitly. Furthermore, the solution space varies depending on the algorithm that is used to fit the model. If we further assume that *b*_*i*_=*k*_*ib*__1_ for constants *k*_2_,…,*k*_*q*_ then Σ_*XY*_ becomes a rank 1 matrix and these challenges are reduced, thereby leading to a setting where we can start to understand characteristics of multivariate PLS.

Condition 2 and assumption 3 below recapitulate these assumptions where the set of regression coefficients *b*_1_,*b*_2_,…,*b*_*q*_ are represented by the coefficient matrix *B*.

*Condition 2*. There are eigenvectors *v*_*j*_(*j*=1,…,*K*) of Σ_*XX*_ corresponding to different eigenvalues, such that 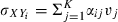 and *α*_*i*1_,…,*α*_*iK*_ are non-zero for *i*=1,…,*q*.

*Assumption 3*. Assume that *Y*=*XB*+*F*, where columns of *F* are independent and from 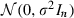. *B* is a rank 1 matrix with singular value decomposition *ϑuv*^T^, where *ϑ* denotes the singular value and *u* and *v* are left and right singular vectors respectively. In addition, *ϑ*<∞ and *q* is fixed.

Lemma 1 proves the convergence of the first direction vector which plays a key role in forming the solution space of the PLS estimator. The proof is provided in Appendix A.

*Lemma 1*. Under assumption 3, 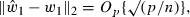 where 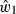 is the estimate of the first direction vector *w*_1_ and is given by Σ_*XX*_*u*/‖Σ_*XX*_*u*‖_2_.

The main implication of lemma 1 is that, under the given conditions, the convergence rate of the first direction vector from multivariate PLS is the same as that of a single univariate PLS. Since the application of univariate PLS for a multivariate response requires estimating *q* numbers of separate direction vectors, the advantage of multivariate PLS is immediate. The proof of lemma 1 relies on obtaining the left singular vector *s* by the rank 1 approximation of *S*_*XY*_, minimizing 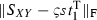. Here, ‖·‖_F_ denotes Frobenius norm, *ς* is the non-zero singular value of *S*_*XY*_ and *s* and *t*_1_ are left and right singular vectors respectively. As a result, *s* can be represented by 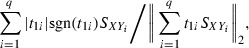 where *t*_1*i*_ is the *i*th element of *t*_1_, and sgn(*t*_1*i*_)=sgn(*s*^T^*S*_*XY*_*i*__). This form of *s* provides intuition for estimating the first multivariate PLS direction vector. Namely, the first direction vector can be interpreted as the weighted sum of sign-adjusted covariance vectors. Directions with stronger signals contribute more in a sign-adjusted manner.

The above discussion highlighted the advantage of multivariate PLS compared with univariate PLS in terms of estimation of the direction vectors. Next, we present the convergence result of the final PLS solution.

*Theorem 2*. Under assumptions 1 and 3, condition 2 and for fixed *K* and *q*, 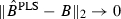 in probability if and only if *p*/*n*→0.

Theorem 2 implies that, under the given conditions and for fixed *K* and *q*, the PLS estimator is consistent regardless of the algorithmic variant that is used if *p*/*n*→0. Although PLS solutions from algorithmic variants might differ for finite *n*, these solutions are consistent. Moreover, the fixed *q* case is practical in most applications because we can always cluster *Y*s into smaller groups before linking them to *X*. We refer to Chun and Keleş (2009) for an application of this idea within the context of expression quantitative loci mapping.

Our results for multivariate *Y* are based on the equal variance assumption on the components of the error matrix *F*. Even though the popular objective functions of multivariate PLS given in expressions (2) and (3) do not involve a scaling factor for each component of multivariate *Y*, in practice, *Y*s are often scaled before the analysis. Violation of the equal variance assumption will affect the performance of PLS regression (Helland, 2000). Therefore, if there are reasons to believe that the error levels in *Y*, not the signal strengths, are different, scaling will aid in satisfying the equal variance assumption of our theoretical result.

### 2.3. Motivation for the sparsity principle in partial least squares regression

To motivate the sparsity principle, we now explicitly illustrate how a large number of irrelevant variables affect the PLS estimator through a simple example. This observation is central to our methodological development. We utilize the closed form solution of Helland (1990) for univariate PLS regression 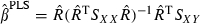, where 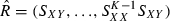.

Assume that *X* is partitioned into (*X*_1_,*X*_2_), where *X*_1_ and *X*_2_ denote *p*_1_ relevant and *p*−*p*_1_ irrelevant variables respectively and each column of *X*_2_ follows 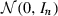. We assume the existence of a latent variable (*K*=1) as well as a fixed number of relevant variables (*p*_1_) and let *p* grow at the rate *O*(*k*^′^*n*), where the constant *k*^′^ is sufficiently large to have 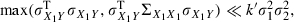
(4) where *σ*_1_ and *σ*_2_ are from Section 2.2.1.

It is not difficult to obtain a sufficiently large *k*^′^ to satisfy condition (4) for fixed *p*_1_. Then, the PLS estimator can be approximated by 
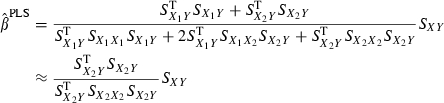
(5)


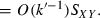
(6)

Approximation (5) follows from lemma 2 in Appendix A and assumption (4). Approximation (6) is due to the fact that the largest and smallest eigenvalues of the Wishart matrix are *O*(*k*^′^) (Geman, 1980). In this example, the large number of noise variables forces the loadings in the direction of *S*_*XY*_ to be attenuated and thereby cause inconsistency.

From a practical point of view, since latent factors of PLS have contributions from all the variables, the interpretation becomes difficult in the presence of large numbers of noise variables. Motivated by the observation that noise variables enter the PLS regression via direction vectors and attenuate estimates of the regression parameters, we consider imposing sparsity on the direction vectors.

## 3. Sparse partial least squares regression

### 3.1. Finding the first sparse partial least squares direction vector

We start with formulation of the first spls direction vector and illustrate the main ideas within this simpler problem. We formulate the objective function for the first spls direction vector by adding an *L*_1_-constraint to problems (2) and (3): 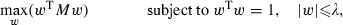
(7) where *M*=*X*^T^*YY*^T^*X* and *λ* determines the amount of sparsity. The same approach has been used in SPCA. By specifying *M* to be *X*^T^*X* in expression (7), this objective function coincides with that of a simplified component lasso technique called ‘SCOTLASS’ (Jolliffe *et al.*, 2003) and both SPLS and SPCA correspond to the same class of maximum eigenvalue problem with a sparsity constraint.

Jolliffe *et al.* (2003) pointed out that the solution of this formulation tends not to be sufficiently sparse and the problem is not convex. This convexity issue was revisited by d'Aspremont *et al.* (2007) in direct SPCA by reformulating the criterion in terms of *W*=*ww*^T^, thereby producing a semidefinite programming problem that is known to be convex. However, the sparsity issue remained.

To obtain a sufficiently sparse solution, we reformulate the SPLS criterion (7) by generalizing the regression formulation of SPCA (Zou *et al.*, 2006). This formulation promotes the exact zero property by imposing an *L*_1_-penalty onto a surrogate of the direction vector (*c*) instead of the original direction vector (*w*), while keeping *w* and *c* close to each other: 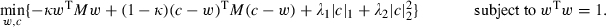
(8)

In this formulation, the *L*_1_-penalty encourages sparsity on *c* whereas the *L*_2_-penalty addresses the potential singularity in *M* when solving for *c*. We shall rescale *c* to have norm 1 and use this scaled version as the estimated direction vector. We note that this problem becomes that of SCOTLASS when *w*=*c* and *M*=*X*^T^*X*, SPCA when 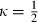 and *M*=*X*^T^*X*, and the original maximum eigenvalue problem of PLS when *κ*=1. We aim to reduce the effect of the concave part (hence the local solution issue) by using a small *κ*.

### 3.2. Solution for the generalized regression formulation of sparse partial least squares

We solve the generalized regression formulation of SPLS given in expression (8) by alternatively iterating between solving for *w* for fixed *c* and solving for *c* after fixing *w*.

For the problem of solving *w* for fixed *c*, the objective function in problem (8) becomes 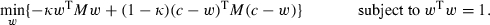
(9)

For 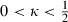, problem (9) can be rewritten as 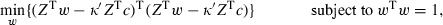 where *Z*=*X*^T^*Y* and *κ*^′^=(1−*κ*)/(1−2*κ*). This constrained least squares problem can be solved via the method of Lagrange multipliers and the solution is given by *w*=*κ*^′^(*M*+λ^*^I)^−1^*Mc* where the multiplier λ^*^ is the solution of *c*^T^*M*(*M*+*λI*)^−2^*Mc*=*κ*^′2^. For 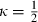, the objective function in problem (9) reduces to −*w*^T^*Mc* and the solution is *w*=*UV*^T^, where *U* and *V* are obtained from the singular value decomposition of *Mc* (Zou *et al.*, 2006).

When solving for *c* for fixed *w*, problem (8) becomes 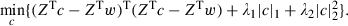
(10)

This problem, which is equivalent to the naive elastic net (EN) problem of Zou and Hastie (2005) when *Y* in the naive EN is replaced with *Z*^T^*w*, can be solved efficiently via the least angle regression spline algorithm LARS (Efron *et al.*, 2004). SPLS often requires a large *λ*_2_-value to solve problem (10) because *Z* is a *q*×*p* matrix with usually small *q*, i.e. *q*=1 for univariate *Y*. As a remedy, we use an EN formulation with *λ*_2_=∞ and this yields the solution to have the form of a soft thresholded estimator (Zou and Hastie, 2005). This concludes our solution of the regression formulation for general *Y* (univariate or multivariate). We further have the following simplification for univariate *Y* (*q*=1).

*Theorem 3*. For univariate *Y*, the solution of problem (8) is 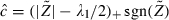, where 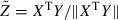 is the first direction vector of PLS.

*Proof*. For a given *c* and *κ*=0.5, it follows that 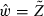 since the singular value decomposition of *ZZ*^T^*c* yields 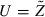 and *V*=1. For a given *c* and 0<*κ*<0.5, the solution is given by *w*={*Z*^T^*c*/(‖*Z*‖^2^+λ^*^)}*Z* by using the Woodbury formula (Golub and van Loan, 1987). Noting that *Z*^T^*c*/(‖*Z*‖^2^+λ^*^) is a scalar and by the norm constraint, we have 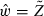. Since 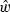 does not depend on *c*, we have 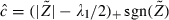 for large *λ*_2_.

## 4. Implementation and algorithmic details

### 4.1. Sparse partial least squares algorithm

In this section, we present the complete SPLS algorithm which encompasses the formulation of the first SPLS direction vector from Section 3.1 as well as an efficient algorithm for obtaining all the other direction vectors and coefficient estimates.

In principle, the objective function for the first SPLS direction vector can be utilized at each step of the NIPALS or SIMPLS algorithm to obtain the rest of the direction vectors. We call this idea the naive SPLS algorithm. However, this naive SPLS algorithm loses the conjugacy of the direction vectors. A similar issue appears in SPCA, where none of the methods proposed (Jolliffe *et al.*, 2003; Zou *et al.*, 2006; d'Aspremont *et al.*, 2007) produces orthogonal sparse principal components. Although conjugacy can be obtained by the Gram–Schmidt conjugation of the derived sparse direction vectors, these post-conjugated vectors do not inherit the property of Krylov subsequences which is known to be crucial for the convergence of the algorithm (Krämer, 2007). Essentially, such a post-orthogonalization does not guarantee the existence of the solution among the iterations.

To address this concern, we propose an SPLS algorithm which leads to a sparse solution by keeping the Krylov subsequence structure of the direction vectors in a restricted *X*-space of selected variables. Specifically, at each step of either the NIPALS or the SIMPLS algorithm, it searches for relevant variables, the so-called active variables, by optimizing expression (8) and updates all direction vectors to form a Krylov subsequence on the subspace of the active variables. This is simply achieved by conducting PLS regression by using the selected variables. Let 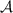 be an index set for active variables and *K* the number of components. Denote 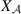 as the submatrix of *X* whose column indices are contained in 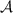. The SPLS algorithm can utilize either the NIPALS or the SIMPLS algorithm as described below.

Step 1: set 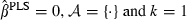. For the NIPALS algorithm set, *Y*_1_=*Y*, and for the SIMPLS algorithm set *X*_1_=*X*.
Step 2: while *k*≤*K*,

- find 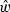 by solving the objective (8) in Section 3.1 with 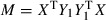 for the NIPALS and 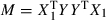 for the SIMPLS algorithm,
- update 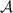 as 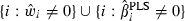,
- fit PLS with 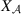 by using *k* number of latent components and
- update 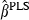 by using the new PLS estimates of the direction vectors, update *k* with *k*←*k*+1, for the NIPALS algorithm, update *Y*_1_ through 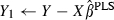 and for the SIMPLS algorithm, update *X*_1_ through 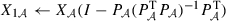, where 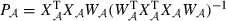.

The original NIPALS algorithm includes deflation steps for both *X*- and *Y*- matrices, but the same *M*-matrix can be computed via the deflation of either *X* or *Y* owing to the idempotency of the projection matrix. In our SPLS–NIPALS algorithm, we chose to deflate the *Y*-matrix because, in that case, the eigenvector *X*^T^*Y*_1_/‖*X*^T^*Y*_1_‖ of *M* is proportional to the current correlations in the LARS algorithm for univariate *Y*. Hence, the LARS and SPLS–NIPALS algorithms use the same criterion to select active variables in this case. However, the SPLS–NIPALS algorithm differs from LARS in that it selects more than one variable at a time and utilizes the conjugate gradient (CG) method to compute the coefficients at each step (Friedman and Popescu, 2004). This, in particular, implies that the SPLS–NIPALS algorithm can select a group of correlated variables simultaneously. The cost of computing coefficients at each step of the SPLS algorithm is less than or equal to that of LARS as the CG method avoids matrix inversion.

The SPLS–SIMPLS algorithm has similar attributes to the SPLS–NIPALS algorithm. It also uses the CG method and selects more than one variable at each step and handles multivariate responses. However, the *M*-matrix is no longer proportional to the current correlations of the LARS algorithm. SIMPLS yields direction vectors directly satisfying the conjugacy constraint, which may hamper the ability of revealing relevant variables. In contrast, the direction vectors at each step of the NIPALS algorithm are derived to maximize the current correlations on the basis of residual matrices, and conjugated direction vectors are computed at the final stage. Thus, the SPLS–NIPALS algorithm is more likely to choose the correct set of relevant variables when the signals of the relevant variables are weak. A small simulation study investigating this point is presented in Section 5.1.

### 4.2. Choosing the thresholding parameter and the number of hidden components

Although the SPLS regression formulation in expression (8) has four tuning parameters (*κ*,*λ*_1_,*λ*_2_ and *K*), only two of these are key tuning parameters, namely the thresholding parameter *λ*_1_ and the number of hidden components *K*. As we discussed in theorem 3 of Section 3.2, the solution does not depend on *κ* for univariate *Y*. For multivariate *Y*, we show with a simulation study in Section 5.2 that setting *κ* smaller than 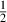 generally avoids local solution issues. Different *κ*-values have the effect of starting the algorithm with different starting values. Since the algorithm is computationally inexpensive (the average run time including the tuning is only 9 min for a sample size of *n*=100 with *p*=5000 predictors on a 64-bit machine with 2.66 GHz central processor unit), users are encouraged to try several *κ*-values. Finally, as described in Section 3.2, setting the *λ*_2_-parameter to ∞ yields the thresholded estimator which depends only on *λ*_1_. Therefore, we proceed with the tuning mechanisms for the two key parameters *λ*_1_ and *K*. We start with univariate *Y* since imposing an *L*_1_-penalty has the simple form of thresholding, and then we discuss multivariate *Y*.

We start with describing a form of soft thresholded direction vector 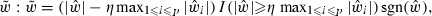 where 0≤*η*≤1. Here, *η* plays the role of the sparsity parameter *λ*_1_ in theorem 3. This form of soft thresholding retains components that are greater than some fraction of the maximum component. A similar approach was utilized in Friedman and Popescu (2004) with hard thresholding as opposed to our soft thresholding scheme. The single tuning parameter *η* is tuned by cross-validation (CV) for all the direction vectors. We do not use separate sparsity parameters for individual directions because tuning multiple parameters is computationally prohibitive and may not produce a unique minimum for the CV criterion.

Next, we describe a hard thresholding approach by the control of the false discovery rate FDR. SPLS selects variables which exhibit high correlations with *Y* in the first step and adds additional variables with high partial correlations in the subsequent steps. Although we are imposing sparsity on direction vectors via an *L*_1_-penalty, the thresholded form of our solution for univariate *Y* allows us to compare and contrast our approach directly with the supervised PC approach of Bair *et al.* (2006) that operates by an initial screening of the predictor variables. Selecting related variables on the basis of correlations has been utilized in supervised PCs, and, in a way, we further extend this approach by utilizing partial correlations in the later steps. Owing to uniform consistency of correlations (or partial correlations after taking into account the effect of relevant variables), FDR control is expected to work well even in the large *p* and small *n* scenario (Kosorok and Ma, 2007). As we described in Section 4, the components of the direction vectors for univariate *Y* have the form of a correlation coefficient (or a partial correlation coefficient after the first step) between the individual covariate and response, and a thresholding parameter can be determined by control of the FDR at a prespecified level *α*. Let 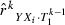 denote the sample partial correlation of the *i*th variable *X*_*i*_ with *Y* given 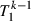, where 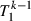 denotes the set of first *k*−1 latent variables included in the model. Under the normality assumption on *X* and *Y*, and the null hypothesis 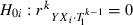, the *z*-transformed (partial) correlation coefficients have the distribution (Bendel and Afifi, 1976) 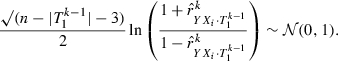


We compute the corresponding *p*-values 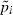, for *i*=1,…,*p*, for the (partial) correlation coefficients by using this statistic and arrange them in ascending order: 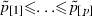. After defining 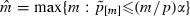, the hard thresholded direction vector becomes 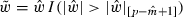 based on the Benjamini and Hochberg (1995) FDR procedure.

We remark that the solution from FDR control is minimax optimal if 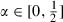 and *α*>*γ*/ log (*p*)(*γ*>0) under independence among tests. As long as *α* decreases with an appropriate rate as *p* increases, thresholding by FDR control is optimal without knowing the level of sparsity and, hence, reduces computation considerably. Although we do not have this independence, this adaptivity may work since the argument for minimax optimality mainly depends on marginal properties (Abramovich *et al.*, 2006).

As discussed in Section 3.2, for multivariate *Y*, the solution for SPLS is obtained through iterations and the resulting solution has a form of soft thresholding. Although hard thresholding with FDR control is no longer applicable, we can still employ soft thresholding based on CV. The number of hidden components, *K*, is tuned by CV as in the original PLS. We note that CV will be a function of two arguments for soft thresholding and that of one argument for hard thresholding and thereby making hard thresholding computationally much cheaper than soft thresholding.

## 5. Simulation studies

### 5.1. Comparison between SPLS–NIPALS and SPLS–SIMPLS algorithms

We conducted a small simulation study to compare variable selection performances of the two SPLS variants, SPLS–NIPALS and SPLS–SIMPLS. The data-generating mechanism is set as follows. Columns of *X* are generated by *X*_*i*_=*H*_*j*_+*ɛ*_*i*_ for *n*_*j*−1_+1≤*i*≤*n*_*j*_, where *j*=1,…,3 and (*n*_0_,*n*_1_,*n*_2_,*n*_3_)=(0,6,13,30). Here, *H*_1_,*H*_2_ and *H*_3_ are independent random vectors from 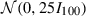 and the *ɛ*_*i*_s are from 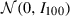. Columns of *Y* are generated by *Y*_1_=0.1*H*_1_−2*H*_2_+*f*_1_, and *Y*_*i*+1_=1.2*Y*_*i*_+*f*_*i*_, where the *f*_*i*_s are from 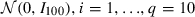. We generated 100 simulated data sets and analysed them using both the SPLS–NIPALS and the SPLS–SIMPLS algorithms. Table 1 reports the first quartile, median, and the third quartile of the numbers of correctly and incorrectly selected variables. We observe that the SPLS–NIPALS algorithm performs better in identifying larger numbers of correct variables with a smaller number of false positive results compared with the SPLS–SIMPLS algorithm. Further investigation reveals that the relevant variables that the SPLS–SIMPLS algorithm misses are typically from the *H*_1_-component with weaker signal.

**Table 1:** Variable selection performances of SPLS–NIPALS *versus* SPLS–SIMPLS algorithms

*Method*	*Number of correct variables*†	*Number of incorrect variables*†
SPLS–NIPALS	9.75 / 12 / 13	0 / 0 / 2
SPLS–SIMPLS	7 / 9 / 13	0 / 2 / 5

† First quartile/median/third quartile.

### 5.2. Setting the weight factor *κ* in the general regression formulation of problem (8)

We ran a small simulation study to examine how the generalization of the regression formulation given in expression (8) helps to avoid the local solution issue. The data-generating mechanism is set as follows. Columns of *X* are generated by *X*_*i*_=*H*_*j*_+*ɛ*_*i*_ for *n*_*j*−1_+1≤*i*≤*n*_*j*_, where *j*=1,…,4 and (*n*_0_,…,*n*_4_)=(0,4,8,10,100). Here, *H*_1_ is a random vector from 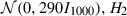 is a random vector from 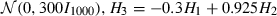 and *H*_4_=0. The *ɛ*_*i*_s are independent identically distributed random vectors from 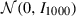. For illustration, we use *M*=*X*^T^*X*. When *κ*=0.5, the algorithm becomes stuck at a local solution in 27 out of 100 simulation runs. When *κ*=0.1,0.3,0.4, the correct solution is obtained in all runs. This indicates that a slight imbalance giving less weight to the concave objective function of formulation (8) might lead to a numerically easier optimization problem.

### 5.3. Comparisons with recent variable selection methods in terms of prediction power and variable selection

In this section, we compare SPLS regression with other popular methods in terms of prediction and variable selection performances in various correlated covariates settings. We include OLS and the lasso, which are not particularly tailored for correlated variables. We also consider dimension reduction methods such as PLS, principal component regression (PCR) and supervised PCs, which ought to be appropriate for highly correlated variables. The EN is also included in these comparisons since it can handle highly correlated variables.

We first consider the case where there is a reasonable number of observations (i.e. *n*>*p*) and set *n*=400 and *p*=40. We vary the number of spurious variables as *q*=10 and *q*=30, and the noise-to-signal ratios as 0.1 and 0.2. Hidden variables *H*_1_,…,*H*_3_ are from 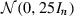, and the columns of the covariate matrix *X* are generated by *X*_*i*_=*H*_*j*_+*ɛ*_*i*_ for *n*_*j*−1_+1≤*i*≤*n*_*j*_, where *j*=1,…,3,(*n*_0_,…,*n*_3_)=(0,(*p*−*q*)/2,*p*−*q*,*p*) and *ɛ*_1_,…,*ɛ*_*p*_ are drawn independently from 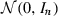. *Y* is generated by 3*H*_1_−4*H*_2_+*f*, where *f* is normally distributed with mean 0. This mechanism generates covariates, subsets of which are highly correlated.

We, then, consider the case where the sample size is smaller than the number of the variables (i.e. *n*<*p*) and set *n*=40 and *p*=80. The numbers of spurious variables are set to *q*=20 and *q*=40, and noise-to-signal ratios to 0.1 and 0.2 respectively. *X* and *Y* are generated similarly to the above *n*>*p* case.

We select the optimal tuning parameters for most of the methods by using tenfold CV. Since the CV curve tends to be flat in this simulation study, we first identify parameters of which CV scores are less than 1.1 times the minimum of the CV scores. We select the smallest *K* and the largest *η* among the selected parameters for SPLS, the largest *λ*_2_ and the smallest step size for the EN and the smallest step size for the lasso. We use the *F*-statistic (the default CV score in the R package ) from the fitted model as a CV score for supervised PC. Then, we use the same procedure to generate an independent test data set and predict *Y* on this test data set on the basis of the fitted models. For each parameter setting, we perform 30 runs of simulations and compute the mean and standard deviation of the mean-squared prediction errors. The averages of the sensitivities and specificities are computed across the simulations to compare the accuracy of variable selection. The results are presented in Tables 2 and 3.

**Table 2:** Mean-squared prediction error for simulations I and II†

*p*/*n*/*q*/*nssettings*	*Mean-squared prediction errors for the following methods:*							
	*PLS (SE)*	*PCR (SE)*	*OLS (SE)*	*Lasso (SE)*	*SPLS1 (SE)*	*SPLS2 (SE)*	*Supervised PCs (SE)*	*EN (SE)*
40/400/10/0.1	31417.9	15717.1	31444.4	208.3	199.8	201.4	198.6	200.1
	(552.5)	(224.2)	(554.0)	(10.4)	(9.0)	(11.2)	(9.5)	(10.0)
40/400/10/0.2	31872.0	16186.5	31956.9	697.3	661.4	658.7	658.8	685.5
	(544.4)	(231.4)	(548.9)	(15.7)	(13.9)	(15.7)	(14.2)	(17.7)
40/400/30/0.1	31409.1	20914.2	31431.7	205.0	203.3	205.5	202.7	203.1
	(552.5)	(1324.4)	(554.2)	(9.5)	(10.1)	(11.1)	(9.4)	(9.7)
40/400/30/0.2	31863.7	21336.0	31939.3	678.6	661.2	663.5	663.5	684.9
	(544.1)	(1307.6)	(549.1)	(13.6)	(14.4)	(15.6)	(14.4)	(19.3)
80/40/20/0.1	29121.4	15678.0		485.2	538.4	494.6	720.0	533.9
	(1583.2)	(652.9)		(48.4)	(70.5)	(63.0)	(240.0)	(75.3)
80/40/20/0.2	30766.9	16386.5		1099.2	1019.5	965.5	2015.8	1050.7
	(1386.0)	(636.8)		(86.0)	(74.6)	(74.7)	(523.6)	(84.5)
80/40/40/0.1	29116.2	17416.1		502.4	506.9	497.7	522.7	545.3
	(1591.7)	(924.2)		(54.0)	(66.9)	(62.8)	(69.4)	(77.1)
80/40/40/0.2	29732.4	17940.8		1007.2	1013.3	964.4	1080.6	1018.7
	(1605.8)	(932.2)		(82.9)	(78.7)	(74.6)	(165.6)	(74.9)

† *p*, the number of covariates; *n*, the sample size; *q*, the number of spurious variables; ns, noise-to-signal ratio; SPLS1, SPLS tuned by FDR control (FDR = 0.1); SPLS2, SPLS tuned by CV; SE, standard error.

Although not so surprising, the methods with an intrinsic variable selection property show smaller prediction errors compared with the methods lacking this property. For *n*>*p*, the lasso, SPLS, supervised PCs and the EN show similar prediction performances in all four scenarios. This holds for the *n*<*p* case, except that supervised PC shows a slight increase in prediction error for dense models (*p*=80 and *q*=20). For the model selection accuracy, SPLS, supervised PCs and the EN show excellent performances, whereas the lasso exhibits poor performance by missing relevant variables. SPLS performs better than other methods for *n*<*p* and high noise-to-signal ratio scenarios. We observe that the EN misses relevant variables in the *n*<*p* scenario, even though its *L*_2_-penalty aims to handle these cases specifically. Moreover, the EN performs well for the right size of the regularization parameter *λ*_2_, but finding the optimal size objectively through CV seems to be a challenging task.

In general, both SPLS–CV and SPLS–FDR perform at least as well as other methods (Table 3). Especially, when *n*<*p*, the lasso fails to identify important variables, whereas SPLS regression succeeds. This is because, although the number of SPLS latent components is limited by *n*, the actual number of variables that makes up the latent components can exceed *n*.

**Table 3:** Model accuracy for simulations I and II†

*p*/*n*/*q*/*ns settings*	*Results for the following methods:*									
	*Lasso*		*SPLS1*		*SPLS2*		*SuperPC*		*EN*	
	*Sensitivity*	*Specificity*	*Sensitivity*	*Specificity*	*Sensitivity*	*Specificity*	*Sensitivity*	*Specificity*	*Sensitivity*	*Specificity*
40/400/10/0.1	0.76	1.00	1.00	0.83	1.00	1.00	1.00	1.00	1.00	0.95
40/400/10/0.2	0.67	1.00	1.00	0.80	1.00	1.00	1.00	1.00	0.94	0.97
40/400/30/0.1	1.00	0.98	1.00	0.83	1.00	1.00	1.00	1.00	1.00	0.95
40/400/30/0.2	0.96	1.00	1.00	0.80	1.00	1.00	1.00	1.00	1.00	0.95
80/40/20/0.1	0.15	1.00	1.00	0.80	1.00	1.00	0.97	0.93	0.72	0.99
80/40/20/0.2	0.12	1.00	1.00	0.67	1.00	1.00	0.86	0.83	0.80	0.98
80/40/40/0.1	0.21	1.00	1.00	0.80	1.00	1.00	1.00	0.93	0.72	0.99
80/40/40/0.2	0.15	1.00	1.00	0.80	1.00	1.00	0.97	0.90	0.80	0.98

† *p*, the number of covariates; *n*, the sample size; *q*, the number of spurious variables; ns, noise-to-signal ratio; SPLS1, SPLS tuned by FDR control (FDR = 0.1); SPLS2, SPLS tuned by CV.

### 5.4. Comparisons of predictive power among methods that handle multicollinearity

In this section, we compare SPLS regression with some of the popular methods that handle multicollinearity such as PLS, PCR, ridge regression, a mixed variance–covariance approach, gene shaving (Hastie *et al.*, 2000) and supervised PCs (Bair *et al.*, 2006). These comparisons are motivated by those presented in Bair *et al.* (2006). We compare only prediction performances since all methods except for gene shaving and supervised PCs are not equipped with variable selection. For the dimension reduction methods, we allow only one latent component for a fair comparison.

Throughout these simulations, we set *p*=5000 and *n*=100. All the scenarios follow the general model of *Y*=*Xβ*+*f*, but the underlying data generation for *X* is varying. We devise simulation scenarios where the multicollinearity is due to the presence of one main latent variable (simulations 1 and 2), the presence of multiple latent variables (simulation 3) and the presence of a correlation structure that is not induced by latent variables but some other mechanism (simulation 4). We select the optimal tuning parameters and compute the prediction errors as in Section 5.3. The results are summarized in Table 4.

**Table 4:** Mean-squared prediction errors†

*Method*	*Mean-squared prediction errors for the following simulations:*			
	*Simulation 1*	*Simulation 2*	*Simulation 3*	*Simulation 4*
PCR1	320.67 (8.07)	308.93 (7.13)	241.75 (5.62)	2730.53 (75.82)
PLS1	301.25 (7.32)	292.70 (7.69)	209.19 (4.58)	1748.53 (47.47)
Ridge regression	304.80 (7.47)	296.36 (7.81)	211.59 (4.70)	1723.58 (46.41)
Supervised PC	252.01 (9.71)	248.26 (7.68)	134.90 (3.34)	263.46 (14.98)
SPLS1(FDR)	256.22 (13.82)	246.28 (7.87)	139.01 (3.74)	290.78 (13.29)
SPLS1(CV)	257.40 (9.66)	261.14 (8.11)	120.27 (3.42)	195.63 (7.59)
Mixed variance–covariance	301.05 (7.31)	292.46 (7.67)	209.45 (4.58)	1748.65 (47.58)
Gene shaving	255.60 (9.28)	292.46 (7.67)	119.39 (3.31)	203.46 (7.95)
True	224.13 (5.12)	218.04 (6.80)	96.90 (3.02)	99.12 (2.50)

† PCR1, PCR with one component; PLS1, PLS with one component; SPLS1(FDR), SPLS with one component tuned by FDR control (FDR = 0.4); SPLS1(CV), SPLS with one component tuned by CV; True, true model.

The first simulation scenario is the same as the ‘simple simulation’ that was utilized by Bair *et al.* (2006), where hidden components *H*_1_ and *H*_2_ are defined as follows: *H*_1*j*_ equals 3 for 1≤*j*≤50 and 4 for 51≤*j*≤*n* and *H*_2*j*_=3.5 for 1≤*j*≤*n*. Columns of *X* are generated by *X*_*i*_=*H*_1_+*ɛ*_*i*_ for 1≤*i*≤50 and *H*_2_+*ɛ*_*i*_ for 51≤*i*≤*p*, where *ɛ*_*i*_ are an independent identically distributed random vector from 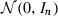. *β* is a *p*×1 vector, where the *i*th element is 1/25 for 1≤*i*≤50 and 0 for 51≤*i*≤*p*. *f* is a random vector from 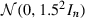. Although this scenario is ideal for supervised PCs in that *Y* is related to one main hidden component, SPLS regression shows a comparable performance with supervised PCs and gene shaving.

The second simulation was referred to as ‘hard simulation’ by Bair *et al.* (2006), where more complicated hidden components are generated, and the rest of the data generation remains the same as in the simple simulation. *H*_1_,…,*H*_5_ are generated by *H*_1*j*_=3 *I*(*j*≤50)+4 *I*(*j*>50),*H*_2*j*_=3.5+1.5 *I*(*u*_1*j*_≤0.4),*H*_3*j*_=3.5+0.5 *I*(*u*_1*j*_≤0.7),*H*_4*j*_=3.5−1.5*I*(*u*_1*j*_≤0.3) and *H*_5*j*_=3.5, for 1≤*j*≤*n*, where *u*_1*j*_,*u*_2*j*_ and *u*_3*j*_ are independent identically distributed random variables from Unif(0,1). Columns of *X* are generated by *X*_*i*_=*H*_*j*_+*ɛ*_*i*_ for *n*_*j*−1_+1≤*i*≤*n*_*j*_, where *j*=1,…,5 and (*n*_0_,…,*n*_5_)=(0,50,100,200,300,*p*). As seen in Table 4, when there are complex latent components, SPLS and supervised PCs show the best performance. These two simulation studies illustrate that both SPLS and supervised PCs have good prediction performances under the latent component model with few relevant variables.

The third simulation is designed to compare the prediction performances of the methods when all methods are allowed to use only one latent component, even though there are more than one hidden components related to *Y*. This scenario aims to illustrate the differences of the derived latent components depending on whether they are guided by the response *Y*. *H*_1_ and *H*_2_ are generated as *H*_1*j*_=2.5 *I*(*j*≤50)+4 *I*(*j*>50),*H*_2*j*_=2.5 *I*(1≤*j*≤25 or 51≤*j*≤75)+4 *I*(26≤*j*≤50 or 76≤*j*≤100). (*H*_3_,…,*H*_6_) are defined in the same way as (*H*_2_,…,*H*_5_) in the second simulation. Columns of *X* are generated by *X*_*i*_=*H*_*j*_+*ɛ*_*i*_ for *n*_*j*−1_+1≤*i*≤*n*_*j*_, *j*=1,…,6, and (*n*_0_,…,*n*_6_)=(0,25,50,100,200,300,*p*). *f* is a random vector from 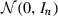. Gene shaving and SPLS both exhibit good predictive performance in this scenario. In a way, when the number of components in the model is fixed, the methods which utilize *Y* when deriving latent components can achieve better predictive performances compared with methods that utilize only *X* when deriving these vectors. This agrees with the prior observation that PLS typically requires a smaller number of latent components than that of PCA (Frank and Friedman, 1993).

The fourth simulation is designed to compare the prediction performances of the methods when the relevant variables are not governed by a latent variable model. We generate the first 50 columns of *X* from a multivariate normal distribution with auto-regressive covariance, and the remaining 4950 columns of *X* are generated from hidden components as before. Five hidden components are generated as follows: *H*_1*j*_ equals 1 for 1≤*j*≤50 and 6 for 51≤*j*≤*n* and *H*_2_,…,*H*_5_ are the same as in the second simulation. Denoting *X*=(*X*^(1)^,*X*^(2)^) by using a partitioned matrix, we generate rows of *X*^(1)^ from 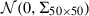, where Σ_50×50_ is from an AR(1) process with an auto-correlation *ρ*=0.9. Columns of *X*^(2)^ are generated by 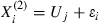 for *n*_*j*−1_+1≤*i*≤*n*_*j*_, where *j*=1,…,5 and (*n*_0_,…,*n*_5_)=(0,50,100,200,300,*p*−50). *β* is a *p*×1 vector and its *i*th element is given by *β*_*i*_=*k*_*j*_ for *n*_*j*−1_+1≤*i*≤*n*_*j*_, where *j*=1,…,6, (*n*_0_,…,*n*_6_)=(0,10,20,30,40,50,*p*) and (*k*_1_,…,*k*_6_)=(8,6,4,2,1,0)/25. SPLS regression and gene shaving perform well, indicating that they have the ability to handle such a correlation structure. As in the third simulation, these two methods may gain some advantage in handling more general correlation structures by utilizing response *Y* when deriving direction vectors.

## 6. Case-study: application to yeast cell cycle data set

Transcription factors (TFs) play an important role for interpreting a genome's regulatory code by binding to specific sequences to induce or repress gene expression. It is of general interest to identify TFs which are related to regulation of the cell cycle, which is one of the fundamental processes in a eukaryotic cell. Recently, Boulesteix and Strimmer (2005) performed an integrative analysis of gene expression and CHIP–chip data measuring the amount of transcription and physical binding of TFs respectively, to address this question. Their analysis focused on estimation rather than variable selection. In this section, we focus on identifying cell cycle regulating TFs.

We utilize a yeast cell cycle gene expression data set from Spellman *et al.* (1998). This experiment measures messenger ribonucleic acid levels every 7 min for 119 min with a total of 18 measurements covering two cell cycle periods. The second data set, CHIP–chip data of Lee *et al.* (2002), contains binding information of 106 TFs which elucidates which transcriptional regulators bind to promoter sequences of genes across the yeast genome. After excluding genes with missing values in either of the experiments, 542 cell-cycle-related genes are retained.

We analyse these data sets with our proposed multivariate (SPLS–NIPALS) and univariate SPLS regression methods, and also with the lasso for a comparison and summarize the results in Table 5. Since CHIP–chip data provide a proxy for the binary outcome of binding, we scale the CHIP–chip data and use tenfold CV for tuning. Multivariate SPLS selects the least number of TFs (32 TFs), and univariate SPLS selects 70 TFs. The lasso selects the largest number of TFs, 100 out of 106. There are a total of 21 experimentally confirmed cell-cycle-related TFs (Wang *et al.*, 2007), and we report the number of confirmed TFs among those selected as a guideline for performance comparisons. In Table 5, we also report a hypergeometric probability calculation quantifying chance occurrences of the number of confirmed TFs among the variables selected by each method. A comparison of these probabilities indicates that multivariate SPLS has more evidence that selection of a large number of confirmed TFs is not due to chance.

**Table 5:** Comparison of the number of selected TFs†

*Method*	*Number of TFs selected (s)*	*Number of confirmed TFs (k)*	*Prob*(*K*≥*k*)
Multivariate SPLS	32	10	0.034
Univariate SPLS	70	17	0.058
Lasso	100	21	0.256
Total	106	21	

† Prob(*K*≥*k*) denotes the probability of observing at least *k* confirmed variables out of 85 unconfirmed and 21 confirmed variables in a random draw of *s* variables.

**Fig. 1:**
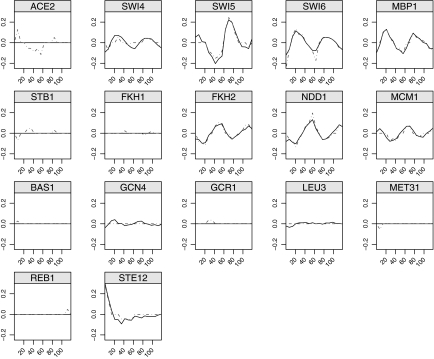
Estimated TF activities for the 21 confirmed TFs (plots for ABF-1, CBF-1, GCR2 and SKN7 are not displayed since the TF activities of the factors were zero by both the univariate and the multivariate SPLS; the *y*-axis denotes estimated coefficients and the *x*-axis is time; multivariate SPLS regression yields smoother estimates and exhibits periodicity): 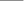, estimated TF activities by the multivariate SPLS regression; 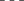, estimated TF activities by univariate SPLS

**Fig. 2:**
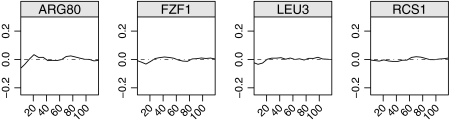
Estimated TF activities selected only by the multivariate SPLS regression; the magnitudes of the estimated TF activities are small but consistent across the time points

We next compare results from multivariate and univariate SPLS. There are a total of 28 TFs which are selected by both methods and nine of these are experimentally verified according to the literature. The estimators, i.e. TF activities, of selected TFs in general show periodicity. This is indeed a desirable property since the 18 time points cover two periods of a cell cycle. Interestingly, as depicted Fig. 1, multivariate SPLS regression obtains smoother estimates of TF activities compared with univariate SPLS. A total of four TFs are selected only by multivariate SPLS regression. These coefficients are small but consistent across the time points (Fig. 2). A total of 42 TFs are selected only by univariate SPLS, and eight of these are among the confirmed TFs. These TFs do not show periodicity or have non zero coefficients only at few time points (the data are not shown). In general, multivariate SPLS regression can capture the weak effects that are consistent across the time points.

## 7. Discussion

PLS regression has been successfully utilized in ill-conditioned linear regression problems that arise in several scientific disciplines. Goutis (1996) showed that PLS yields shrinkage estimators. Butler and Denham (2000) argued that it may provide peculiar shrinkage in the sense that some of the components of the regression coefficient vector may expand instead of shrinking. However, as argued by Rosipal and Krämer (2006), this does not necessarily lead to worse shrinkage because PLS estimators are highly non-linear. We showed that both univariate and multivariate PLS regression estimators are consistent under the latent model assumption with strong restrictions on the number of variables and the sample size. This makes the suitability of PLS for the contemporary very large *p* and small *n* paradigm questionable. We argued and illustrated that imposing sparsity on direction vectors helps to avoid sample size problems in the presence of large numbers of irrelevant variables. We further developed a regression technique called SPLS. SPLS regression is also likely to yield shrinkage estimators since the methodology can be considered as a form of PLS regression on a restricted set of predictors. Analysis of its shrinkage properties is among our current investigations. SPLS regression is computationally efficient since it solves a linear equation by employing a CG algorithm rather than matrix inversion at each step.

We presented the solution of the SPLS criterion for the direction vectors and proposed an accompanying SPLS regression algorithm. Our SPLS regression algorithm has connections to other variable selection algorithms including the EN (Zou and Hastie, 2005) and the threshold gradient (Friedman and Popescu, 2004) method. The EN method deals with collinearity in variable selection by incorporating the ridge regression method into the LARS algorithm. In a way, SPLS handles the same issue by fusing the PLS technique into the LARS algorithm. SPLS can also be related to the threshold gradient method in that both algorithms use only the thresholded gradient and not the Hessian. However, SPLS achieves faster convergence by using the CG.

We presented proof-of-principle simulation studies with combinations of small and large number of predictors and sample sizes. These illustrated that SPLS regression achieves both high predictive power and accuracy for finding the relevant variables. Moreover, it can select a higher number of relevant variables than the available sample size since the number of variables that contribute to the direction vectors is not limited by the sample size.

Our application with SPLS involved two recent genomic data types, namely gene expression data and genomewide binding data of TFs. The response variable was continuous and a linear modelling framework followed naturally. Extensions of SPLS to other modelling frameworks such as generalized linear models and survival models are exciting future directions. Our application with integrative analysis of expression and TF binding date highlighted the use of SPLS within the context of a multivariate response. We expect that several genomic problems with multivariate responses, e.g. linking expression of a cluster of genes to genetic marker data, might lend themselves to the multivariate SPLS framework. We provide an implementation of the SPLS regression methodology as an R package at http://cran.r-project.org/web/packages/spls.

## Appendix A: Proofs of the theorems

We first introduce lemmas 2 and 3 and then utilize these in the proof of theorem 1. ‖*A*‖_2_ for matrix *A* ∈ *R*^*n*×*k*^ is defined as the largest singular value of *A*.

*Lemma 2.* Under assumptions 1 and 2, and *p*/*n*→0,

*Proof.* The first part of lemma 2 was proved by Johnstone and Lu (2004), and we shall show the second part on the basis of their argument. We decompose *S*_*XY*_−*σ*_*XY*_ as (*A*_*n*_+*B*_*n*_+*C*_*n*_)*β*+*D*_*n*_, where  and . We remark that here *E* is defined to be an *n*×*p* matrix of which the *i*th row is *e*_*i*_, whereas the corresponding matrix *Z* in Johnstone and Lu (2004) is a *p*×*n* matrix. We aim to show that the norm of each component of the decomposition is *O*_*p*_{√(*p*/*n*)}. Johnstone and Lu (2004) showed that, if *p*/*n*→*k*_0_ ∈ [0,∞), then ‖*A*_*n*_‖_2_→0,‖*B*_*n*_‖_2_≤*σ*_1_√*k*_0_Σ*ϱ*_*j*_ and  almost surely. Hence, we examine ‖*D*_*n*_‖_2_, components of which have the distributions *υ*^*j*T^*f*=^d^*χ*_*n*_*χ*_1_*U*_*j*_ for 1≤*j*≤*m* and *E*^T^*f*=^d^*χ*_*n*_*χ*_*p*_*U*_*m*+1_, where  and  are *χ*^2^ random variables and the *U*_*j*_s are random vectors, uniform on the surface of the unit sphere *S*^*p*−1^ in *R*^*p*^. After denoting *a*_*j*_=*υ*^*j*T^*f* for 1≤*j*≤*m* and *a*_*m*+1_=*E*^T^*f*, we have that  almost surely, for 1≤*j*≤*m*, and  almost surely from the previous results on the distributions. By using a version of the dominated convergence theorem (Pratt, 1960), the results follow:  almost surely ‖*D*_*n*_‖_2_→√*k*_0_*σ*_1_*σ*_2_ almost surely and  almost surely, and thus the lemma is proved.

*Lemma 3.* Under assumptions 1 and 2 and *p*/*n*→0,

(11)

(12)

*Proof.* Both of these bounds (equations (11) and (12)) are direct consequences of lemma 2. By using the triangular inequality, Hölder's inequality and lemma 2, we have that

for some constants *k*_1_ and *k*_2_ and

### A.1. Proof of theorem 1

We start with proving the first part of theorem 1. We use the closed form solution

where . First, we establish that

By using the triangular inequality and Hölder's inequality,

It is sufficient to show that  and  in probability.

The first claim is proved by using the definition of a matrix norm and lemmas 2 and 3 as

For the second claim, we focus on  since

(Golub and van Loan, 1987). Here, ‖(*R*Σ_*XX*_*R*)^−1^‖_2_ and  are finite as (*R*Σ_*XX*_*R*)^−1^ and  are non-singular for a given *K*. Using this fact as well as the triangular and Hölder's inequalities, we can easily show the second claim. The third claim follows by the fact that  in probability, lemma 2 and the triangular and Hölder's inequalities.

Next, we can establish that  by using the same argument of proposition 1 of Naik and Tsai (2000).

We, now, prove the second part of theorem 1. Since  almost surely,

(13)

If  in probability for *p*/*n*→*k*_0_(>0),

(14)

Since ‖*E*^T^*f*/*n*‖_2_≠0 almost surely, equation (14) implies that  as *n*→∞.

This contradicts the fact that *E*^T^*f*=^d^*χ*_(*n*)_*χ*_(*p*)_*U*_*p*_, where *U*_*p*_ is a vector uniform on the surface of the unit sphere *S*^*p*−1^, as the dimension of  is *p*−*K*.

### A.2. Proof of lemma 1

We remark that , and

from the triangular inequality, proof of theorem 1 and . Then, we have

### A.3. Proof of theorem 2

We start with the sufficient condition of the convergence. We shall first characterize the space that is generated by the direction vectors of each algorithm. For the NIPALS algorithm, we denote  and *D*_*K*_=(*d*_1_,…,*d*_*K*_) as direction vectors for the original covariate and the deflated covariates respectively. The first direction vector  is obtained by *S*_*XY*_*t*_1_/‖*S*_*XY*_*t*_1_‖_2_, where *t*_1_ is the right singular vector of *S*_*XY*_. We denote *s*_*i*,1_=*S*_*XY*_*t*_*i*_/‖*S*_*XY*_*t*_*i*_‖_2_, as this form of vector recurs in the remaining steps. Then, . Define *ψ*_*i*_ as the step size vector at the *i*th step, and the *i*th current correlation matrix  as . The current correlation matrix at the second step is given by  and thus the second direction vector *d*_2_ is proportional to , where *t*_2_ is the right singular vector of . Then , where . Similarly, we can obtain

Now, we observe that  does not form a Krylov space, because *s*_*i*,1_ is not the same as *s*_1,1_ for multivariate *Y*. However, it forms a Krylov space for large *n*, since ‖*S*_*XY*_*t*/‖*S*_*XY*_*t*‖_2_−*w*_1_‖_2_→0 for any *q*-dimensional random vector *t* subject to ‖*t*‖_2_=1 almost surely, following lemma 1.

For the SIMPLS algorithm, using the fact that the *i*th direction vector of SIMPLS is obtained sequentially from the left singular vector of , where , we can characterize

We note that *s*_*i*,1_s and *l*_*i*,*j*_s from the NIPALS and SIMPLS algorithms are different because the *t*_*i*_s are from  and  for the NIPALS and SIMPLS algorithms respectively.

Next, we shall focus on the convergence of the NIPALS estimator, because the convergence of the SIMPLS estimator can be proved by the same argument owing to the structural similarity of  and .

Denoting  and , one can show that  by using the fact ‖*s*_*i*,1_−*w*_1_‖_2_=*O*_*p*_{√(*p*/*n*)} for *i*=1,…,*K*. Since  can also be represented as , we have that . Thus, we now deal with the convergence of , which has a similar form to that of the univariate response case.

Since  for *i*=1,…,*K*, one can show that , where *R*=(*w*_1_,Σ_*XX*_*w*_1_,…,Σ_*XX*_*w*_1_). The convergence of the estimator can be established similarly to the argument in theorem 1 with the following additional argument:

(15)

Inequality (15) follows from observing that each column of the matrix

follows  independently. The remainder of the proof is a simple extension of the proof of theorem 1.

The necessity condition of the convergence is proved as follows. Assume that  in probability, when *p*/*n*→*k*_0_ (>0). Following the argument in the proof of theorem 1, we have

Since ‖*E*^T^*F*/*n*‖_2_≠0 almost surely, this equation implies that  as *n*→∞.

If *p*/*n*→*k*_0_ (>0), this contradicts the fact that *E*^T^*F*_*i*_=^d^*χ*_(*n*)_*χ*_(*p*)_*U*_*p*_, where *F*_*i*_ denotes the *i*th column of *F* and *U*_*p*_ is a vector uniform on the surface of the unit sphere *S*^*p*−1^, as the dimension of  is *p*−*K*.
